# Alpha-synuclein: a pathological factor with Aβ and tau and biomarker in Alzheimer’s disease

**DOI:** 10.1186/s13195-022-01150-0

**Published:** 2022-12-31

**Authors:** Kyu Hwan Shim, Min Ju Kang, Young Chul Youn, Seong Soo A. An, SangYun Kim

**Affiliations:** 1 Department of Bionano Technology, Gachon University, Seongnam-Si, Gyeonggi-Do Republic of Korea; 2 Department of Neurology, Veterans Health Service Medical Center, Veterans Medical Research Institute, Seoul, Republic of Korea; 3 Department of Neurology, Chung-Ang University Hospital, Seoul, Republic of Korea; 4 Department of Neurology, Seoul National University Bundang Hospital and Seoul National University College of Medicine, Seongnam-Si, Gyeonggi-Do Republic of Korea

**Keywords:** Alzheimer’s disease, α-Synuclein, Cerebrospinal fluid, Amyloid-beta, Tau, Biomarker

## Abstract

**Background:**

Alpha-synuclein (α-syn) is considered the main pathophysiological protein component of Lewy bodies in synucleinopathies. α-Syn is an intrinsically disordered protein (IDP), and several types of structural conformations have been reported, depending on environmental factors. Since IDPs may have distinctive functions depending on their structures, α-syn can play different roles and interact with several proteins, including amyloid-beta (Aβ) and tau, in Alzheimer’s disease (AD) and other neurodegenerative disorders.

**Main body:**

In previous studies, α-syn aggregates in AD brains suggested a close relationship between AD and α-syn. In addition, α-syn directly interacts with Aβ and tau, promoting mutual aggregation and exacerbating the cognitive decline. The interaction of α-syn with Aβ and tau presented different consequences depending on the structural forms of the proteins. In AD, α-syn and tau levels in CSF were both elevated and revealed a high positive correlation. Especially, the CSF α-syn concentration was significantly elevated in the early stages of AD. Therefore, it could be a diagnostic marker of AD and help distinguish AD from other neurodegenerative disorders by incorporating other biomarkers.

**Conclusion:**

The overall physiological and pathophysiological functions, structures, and genetics of α-syn in AD are reviewed and summarized. The numerous associations of α-syn with Aβ and tau suggested the significance of α-syn, as a partner of the pathophysiological roles in AD. Understanding the involvements of α-syn in the pathology of Aβ and tau could help address the unresolved issues of AD. In particular, the current status of the CSF α-syn in AD recommends it as an additional biomarker in the panel for AD diagnosis.

## Introduction

Alzheimer’s disease (AD) is characterized by extracellular amyloid-beta (Aβ) plaques and intracellular neurofibrillary or extraneuronal ghost tau tangles in the brain. Recently, the pathophysiology of other proteins, including the triggering receptor expressed on myeloid cells 2, transactive response DNA-binding protein 43, and α-synuclein (α-syn), has drawn attention for their direct and indirect associations in AD. α-Syn is the major constituent protein of Lewy bodies, the hallmark of Parkinson’s disease (PD) [1]. Accumulation of α-syn has been found in the brain of patients with AD, as well as in patients with synucleinopathies, such as PD, dementia with Lewy bodies (DLB), and multiple system atrophy [2]. However, the physiological and pathophysiological structures or functions of α-syn in AD are not fully understood. Furthermore, whether altered α-syn levels are a causal factor or consequential result of AD is unknown.

In 1993, Uéda et al. proposed an amyloidosis mechanism of α-syn and its involvement in the pathogenesis of AD by identifying non-Aβ components (NACs), now known as fragments of α-syn, in Aβ plaques [3]. Immunolabeling using an α-syn antibody verified abundant α-syn at the center of Aβ plaques, confirming the contribution of α-syn in the formation of Aβ plaques [4]. In addition, α-syn-positive inclusions were co-localized with neurofibrillary tangles, suggesting the influence of tau aggregation in Lewy body formation [5]. Previous studies showd that over half of the patients with autopsy-confirmed familial or sporadic AD had comorbid α-syn pathology in addition to Aβ and tau [5–7]. AD patients with autopsy-diagnosed Lewy body variants presented with more rapid cognitive deterioration and a higher mortality rate than patients with pure AD [8]. In contrast, a study showed no correlation of concurrent Lewy body abnormalities in AD with variability in clinical features, including cognitive decline, disease duration, or the presence of hallucinations or extrapyramidal signs [9]. This was supported by a recent autopsy study in which age-associated clinical and cognitive heterogeneities were mediated by the mid-frontal/hippocampal neurofibrillary tangle ratio, not by non-AD pathologies, such as the α-syn co-pathology [10]. On the other hand, patients with autopsy-confirmed quadruple pathologies (Aβ, tau, α-syn, and transactive response DNA-binding protein 43) were associated with a probability of aggressive progression in disease [11].

Although Lewy bodies were most often found in the amygdala and hippocampus of patients with AD, α-syn-positive inclusions, which differ from typical Lewy bodies, were also found [5, 7]. Regional distributions of α-syn and spreads of its pathological patterns differed between typical Lewy body diseases and AD. While the identified pathology of α-syn in DLB and PD spread from the brainstem to the limbic area and the neocortex [12, 13], α-syn in AD with amygdala-predominant Lewy bodies (AD/ALB) may show a spread to lower regions from the upper neuraxis [14]. In addition, immunohistochemical and biochemical analyses of DLB have revealed unique strain-like variations in α-syn pathologies in the amygdala, which are less in AD/ALB [15]. These close associations of α-syn in AD pathology and distinctions from synucleinopathies suggested its possible differential classification of the disease groups depending on the presence of α-syn aggregations [5]. In contrast, despite the absence of Lewy body-related pathology, levels of monomers and oligomers of intracellular soluble α-syn were elevated in the inferior temporal lobe of the AD brain, indicating the importance of the soluble form of α-syn in AD [16, 17]. Furthermore, α-syn levels were elevated in the cerebrospinal fluid (CSF) of patients with AD and are strongly correlated with tau levels [18]. Neuropathologically diagnosed AD patients with α-syn pathology had lower CSF total tau, phosphorylated tau 181, and neurogranin levels, which correlated with elevated α-syn levels [19]. Although the pathophysiological roles of α-syn in AD are unclear, growing evidence suggests that α-syn is directly involved in the pathophysiology of AD.

### Variability in the α-syn structure

**Fig. 1:**
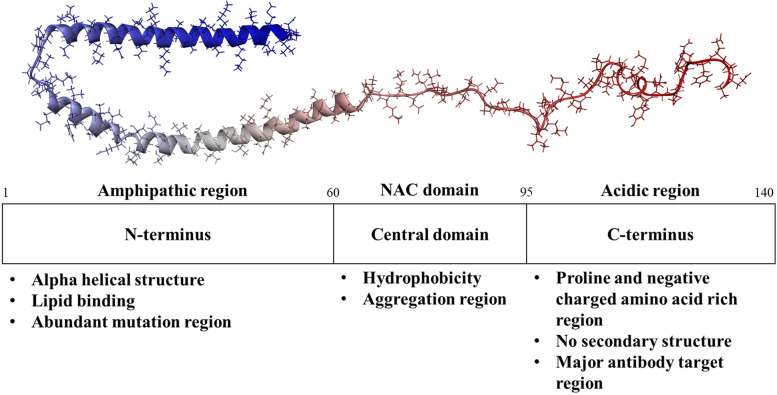
Characteristics of the three regions in α-synuclein. α-syn, alpha-synuclein; NAC, non-amyloid-beta component

α-syn is a small protein composed of 140 amino acids (aa) and classified into three major domains (Fig. 1) [20]. The hydrophilic N-terminal domain (1–60 aa) is an amphipathic domain with an alpha-helical structure that includes the repeated consensus sequence KTKEGV. A characteristic property of this region is its involvement in binding to the lipid membrane [21]. Several missense mutations in this domain, such as A53T, A30P, and E46K, are involved in neurodegenerative disorders, particularly PD [22]. The central domain (61–95 aa) is referred to as the NAC, which can aggregate by forming a beta-sheet structure through its hydrophobic amino acids [3, 23]. The C-terminal domain (96–140 aa) contains abundant proline and strongly negatively charged amino acids, termed acidic tail, without a specific structure [12]. Alternative splicing of the *SNCA* gene transcript results in four protein isoforms; however, their roles under physiological and pathological conditions have not been well elucidated [24].

α-Syn, considered to be an intrinsically disordered protein, can remain in a natively unfolded protein. Purified recombinant α-syn exists as a non-compact mixture of conformers with little secondary structure [25]. Interestingly, α-syn is stabilized by forming an alpha-helix secondary structure when the N-terminal domain is bound to the lipid membrane [23, 26]. The α-helical secondary structure of α-syn reverted to its unfolded conformation upon dissociation from the membrane [27]. However, purified native α-syn from human erythrocytes exists in the form of a tetramer with an α-helical structure, which could resist self-aggregation into protofibril/fibril conformations [28]. In addition, a stable multimeric state of α-syn is formed in the absence of a lipid membrane under non-denaturing conditions on electron micrograph reconstruction or nuclear magnetic resonance (NMR) studies [29]. However, a study showed that identified α-syn in the mouse brain had an unfolded monomeric structure that was prone to aggregation [30]. A review suggested that α-syn could exist in the monomeric structure of the transient state in the cytoplasm and be multimerized through interactions with the membrane until it resumed its physiological functions in the cell [31]. Evidence remains limited for the helical tetramer of α-syn, and further biochemical studies should be conducted to fully understand the physiological and pathophysiological conformations of α-syn in cells. Cryo-electron microscopy (cryo-EM) presented a high-resolution image of α-syn in the fibril state. With full-length recombinant human α-syn, cryo-EM on negative staining showed helical reconstructions, containing two polymorphic fibrils, i.e., rod and twister [32], supporting α-syn fibrils with different lengths extracted from the brain tissue of patients with PD or multiple system atrophy (MSA) [33, 34]. Compared to α-syn fibrils derived from the final product of the protein misfolding cyclic amplification reaction, a technique for amplifying α-syn aggregates, patients with MSA revealed an average shorter twisting distance of α-syn fibrils than those with PD [35]. Brain-derived α-syn fibrils from patients with DLB showed less twisted and thinner structures than those from patients with MSA [36]. Additionally, Peng et al. demonstrated different seeding activities of α-syn fibril strains in the brains with different synucleinopathies [37]. Additional advanced technologies would allow us to demonstrate that the structure of α-syn fibrils could be disease-specific, which could help understand the importance of structural heterogeneity of α-syn in understanding the pathogenicity of the disease.

**Fig. 2:**
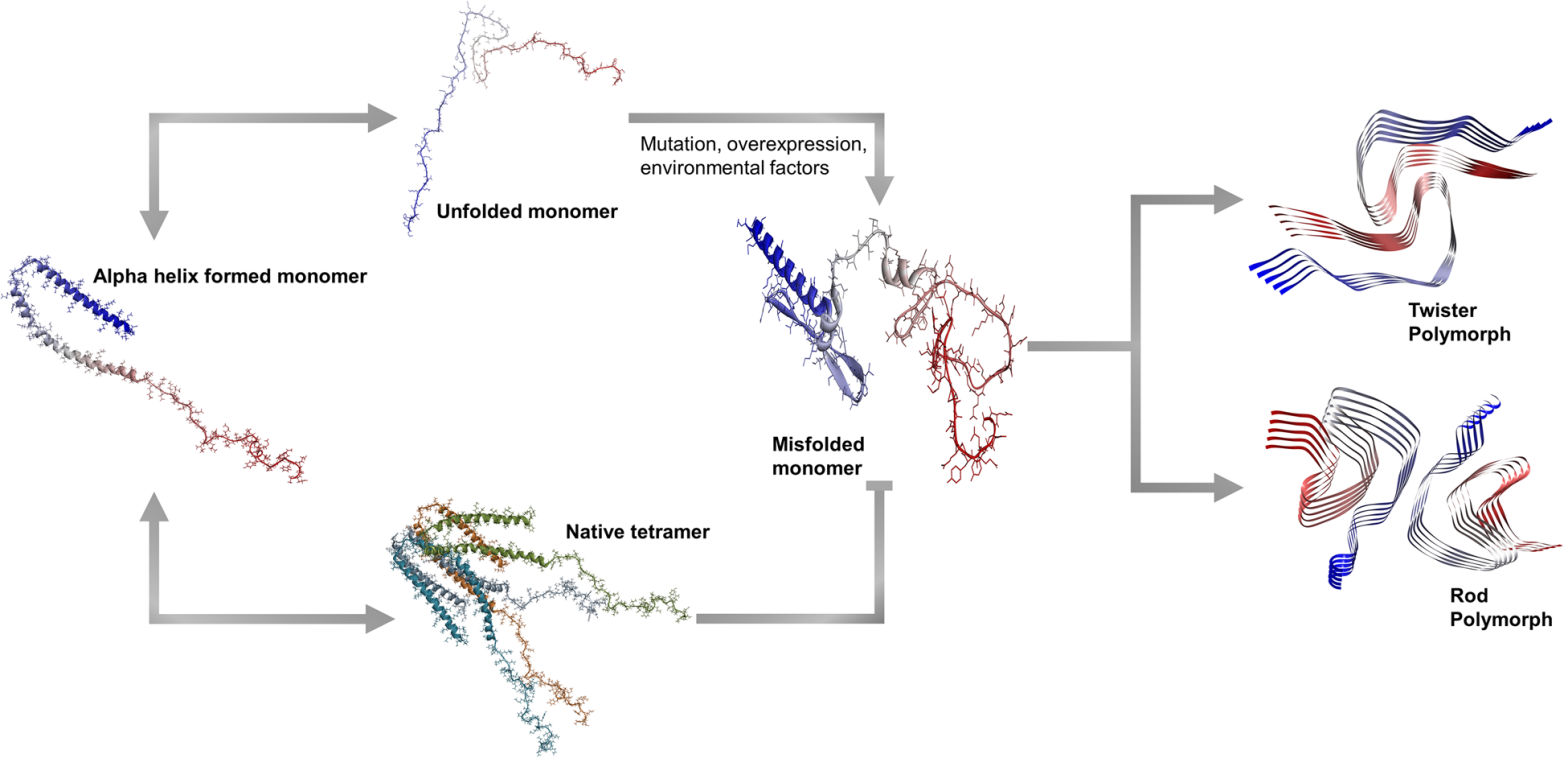
Conformational diversity of α-syn. Modeling of α-synuclein monomer (PDB: 1XQ8), tetramer, and fibril from cryo-electron microscopy for both the twister (PDB: 6CU8) and rod polymorph (PDB: 6CU7). α-syn, alpha-synuclein; cryo-EM, cryo-electron microscopy

Comprehensively, α-syn could be transformed into various conformations depending on the surrounding environment owing to the unstable nature of the structure (Fig. 2). Imbalanced physiological conditions, such as overexpression or mutation, may induce abnormal accumulation and aggregation of α-syn, leading to disease. These conformational diversities of α-syn imply that each α-syn structure may play different roles and/or be involved differently in various neurodegenerative disorders.

### Interactions with AD-related proteins

#### Aβ

Aβ, a crucial peptide in the pathophysiology of AD, is derived from the proteolytic process of the amyloid precursor protein by β- and γ-secretase complexes [38, 39]. Interactions of Aβ with other proteins have been suggested to play a pivotal role in the Aβ pathomechanisms of AD [40]. α-Syn has been investigated as a protein that can directly interact with Aβ [41, 42] (Table 1). Direct interactions between Aβ and α-syn have been shown to promote heterotrophic aggregation and intraneuronal accumulation of α-syn, which may exacerbate neuronal pathologies [43]. Based on co-immunoprecipitation in postmortem samples from the AD and DLB brains, α-syn monomers, dimers, trimers, and pentamers formed complexes with Aβ through interactions between the N-terminus of Aβ and the N- and C-terminus of α-syn [44]. A recent in vitro study confirmed that the interaction of Aβ with α-syn could increase the aggregation rate of α-syn, leading to accelerated fibril formation [45]. In several animal studies, transgenic mice with both hSYN and hAPP exhibited accelerated cognitive decline, motor deficits, and formation of α-syn inclusions in the brain, in which the structures of inclusions were partially fibrillar in a double transgenic mouse model and amorphous in single α-syn transgenic mice [43, 46]. Analogously, α-syn can promote Aβ aggregation, but the exact effects of each conformer are still obscure. For instance, α-syn oligomers could induce the formation of Aβ oligomers and stabilize their cross-β structures, resulting in Aβ fibril-like conformations [47]. Fibril forms of α-syn could accelerate the heterogeneous nucleation pathway of Aβ aggregates, whereas α-syn monomers suppressed Aβ aggregation in the secondary step by binding to Aβ fibrils, indicating that different structural forms of α-syn had distinct effects on Aβ aggregation [48]. Recently, α-syn monomers and oligomers were shown to hamper Aβ fibrillization, enhance oligomerization of Aβ monomers, and stabilize Aβ oligomers [49]. Direct interactions between α-syn and Aβ appear to have different consequences, depending on the structural species/stain. Therefore, subsequent studies should clarify the role of α-syn by investigating the defined components in interactions with AD pathophysiology.

**Table 1:** Interaction between α-synuclein and amyloid-beta

Types of ligands	Types of α-syn	Consequences
Aβ	α-syn	Aβ aggregation↑ [41, 42] α-syn aggregation↑ [43, 45]
Aβ monomer	α-syn monomer	Stabilization of α-syn oligomer [44] Aβ fibrilization↓ and Aβ oligomerization↑ [49]
	α-syn oligomer	Stabilization of α-syn oligomer [44]
	α-syn fibril	Aβ fibrilization↑ [49]
Aβ oligomer	α-syn monomer	Aβ fibrilization↓ and Aβ oligomerization↑ [49]
	α-syn oligomer	Stabilization of Aβ cross-β structure [47] Aβ fibrilization↓ and Aβ oligomerization↑ [49]
	α-syn fibril	Aβ fibrilization↑ [49]
Aβ fibril	α-syn monomer	Second nucleation of Aβ↓ [48]
	α-syn fibril	Second nucleation of Aβ↑ [48, 49]

*α-syn* alpha-synuclein, *Aβ* amyloid-beta

#### Tau

**Fig. 3:**
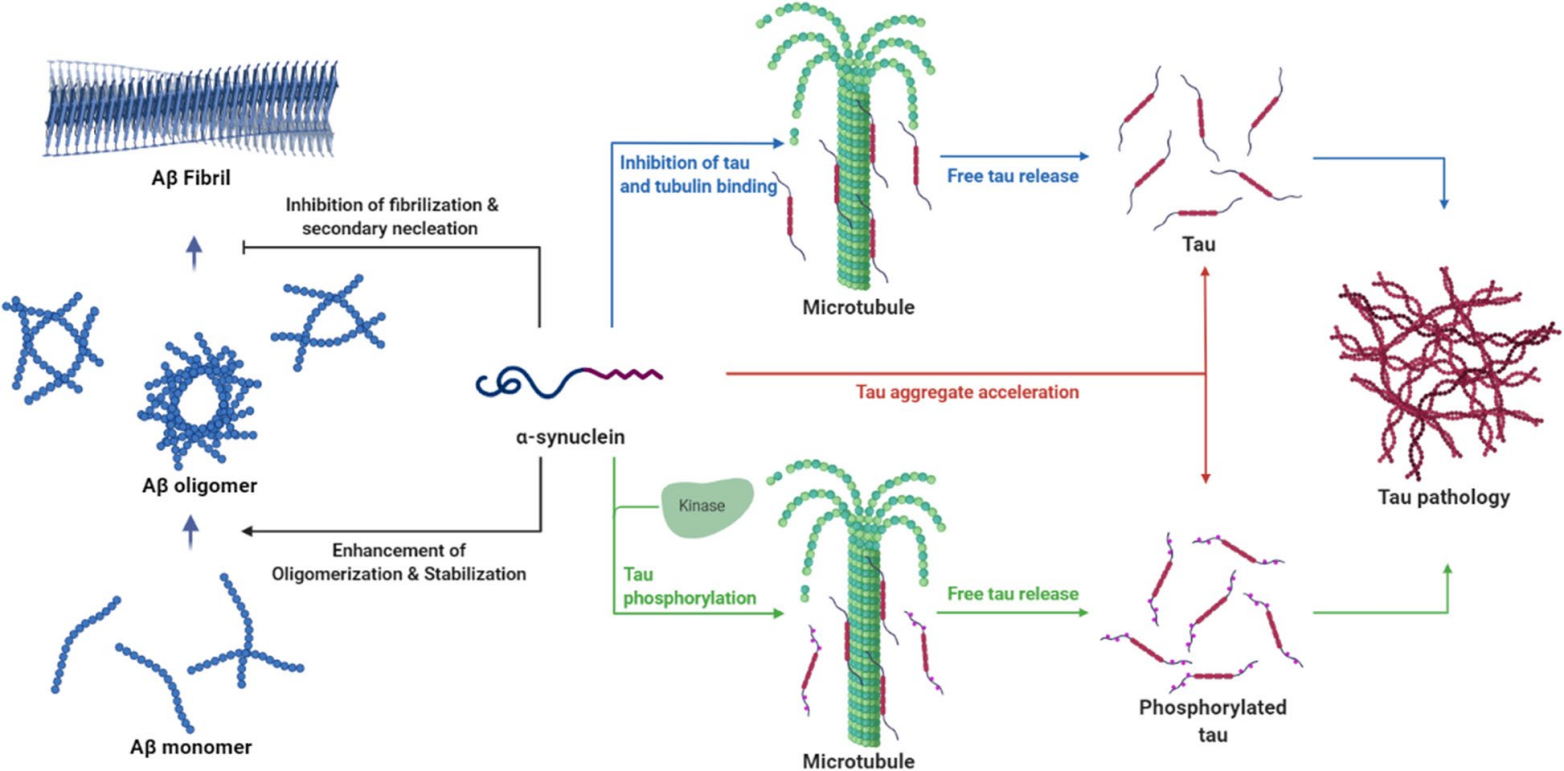
Schematic diagram of the possible relationship of α-synuclein with amyloid-beta, tau, and tubulin α-syn, alpha-synuclein; Aβ, amyloid-beta

Tau is a protein constituting neurofibrillary tangles, a hallmark of AD, and closely related to α-syn and Aβ (Table 2). Tau and α-syn were co-localized in the axons of cells, and their direct interaction in the nerve cell was demonstrated by affinity chromatography of the human brain cytosol [50]. The binding sites were the C-terminus of α-syn (55–140 aa) and the microtubule-binding region of tau (192–383 aa) [50]. This binding led to increased insoluble high-molecular-weight α-syn species and colocalization of tau and α-syn aggregates [51]. Colocalization of tau and α-syn aggregates could be described by prion-like properties of the two proteins that facilitated mutual homogenous/heterogeneous aggregations (Fig. 3). For instance, α-syn could induce tau aggregation; in turn, tau could synergistically accelerate the fibrillization of α-syn [52]. NMR imaging revealed that the monomeric form of tau selectively interacted with the C-terminal region of the α-syn monomer and accelerates α-syn oligomerization and subsequent fibril formation [53]. In addition, α-syn monomers and fibrils promoted tau aggregations [53]. Oikawa et al. suggested different conformational effects of α-syn; only α-syn fibrils, not monomer α-syn, interacted with tau and hampered microtubule assembly by inhibiting the binding of tau to microtubules [54]. α-Syn mutation hampered or enhanced interactions with tau, but the results remain controversial. In in vitro binding assays, α-syn mutations (A30P and A53T) appeared to have no effect on the tau-binding activity [50]. In Förster resonance energy transfer-based analysis, the A30P mutant exhibited a reduced interaction with tau but no effect of A53T or E46K mutants [55]. In cells co-transfected with tau and each mutant α-syn, all mutations (A30P, A53T, and E46K) increased the binding of α-syn with tau and exacerbated the stability of microtubules [56]. In tau mutation (P301L) related to frontotemporal dementia, the reduced binding affinity between the tau mutant and α-syn may promote tau aggregation and higher α-syn fibrils [57]. Treatment with increasing α-syn fibrils also increased tau aggregation by over 50% in P301L-mutant tau cells in comparison with wild-type tau cells, supporting that tau aggregation could result from the interaction with α-syn fibrils, which were accelerated in the P301L-mutant model [58]. Although the direct interaction between α-syn and tau promoted mutual aggregation, additional evidence is required to determine changes in each conformer and mutation determinant.

**Table 2:** Interaction between α-synuclein and tau

Types of ligands	Types of α-syn	Consequences
Tau	α-syn	α-syn aggregation↑ [51–53] Tau phosphorylation↑ [50, 59–62] Tau aggregation↑ [52]
	α-syn fibril	Tau-mediated microtubule assembly↓ [54] Tau phosphorylation↑ [58] Tau aggregation↑ [54, 58]
	A30P α-syn	α-syn and tau interaction↓ [55] or ↑ [56] or no effect [50] Tau-mediated microtubule assembly↓[56]
	E46K α-syn	α-syn and tau interaction↑ [56] or no effect [55] Tau-mediated microtubule assembly↓ [56]
	A53T α-syn	α-syn and tau interaction↑ [56] or no effect [50, 55] Tau-mediated microtubule assembly↓ [56]
P301L Tau	α-syn	α-syn and tau interaction↓ [57] α-syn aggregation↑ [57] Tau aggregation↑ [57]
	α-syn fibril	Tau aggregation↑ [58]

*α-syn* alpha-synuclein

The interaction between these two proteins also promotes tau phosphorylation. The regulation of tau phosphorylation could be accelerated by α-syn, along with several kinases (Fig. 3). Jensen et al. revealed that α-syn increased tau phosphorylation on S262 and S356 residues by protein kinase A up to 66%, depending on the protein concentration ratio [50]. In α-syn-overexpressing mouse models, the existence of phosphorylated tau was associated with the activation of the extracellular signal-regulated and c-Jun N-terminal kinases, which phosphorylated S396 and S404 residues of tau [59]. Hyperphosphorylations of tau at T181, S396, and S404 residues were also induced by activating the tau kinase glycogen synthase kinase-3β (GSK-3β) [60–62]. In the MPP + neurotoxin-induced PD model, α-syn increased the phosphorylation of tau at Ser262, 396, and 404 of tau by forming a heterotrimeric complex with tau and GSK-3β [60, 61]. Moreover, α-syn-deficient cells and α-syn knockout mice showed no change in tau phosphorylation due to the absence of phosphorylated GSK-3β, indicating that tau phosphorylation depended on the presence of α-syn [60, 61]. Under in vitro conditions, α-syn-mediated tau phosphorylation occurred via triple complex formation by the binding of tau to the acidic C-terminus of α-syn and by the interaction between GSK-3β and the NAC and KTEGV domains of α-syn [62]. Tau phosphorylation by this complex was gradually promoted as the α-syn/tau ratio increased, and the molar ratio of tau to α-syn at the maximum point was 1:20 [62]. Consistently, the percentages of phosphorylated tau were increased in the CSF of patients with AD, following increased α-syn/total tau ratio [63]. This accumulated evidence of α-syn-mediated tau phosphorylation suggests that elevated α-syn in AD may promote tau phosphorylation with other kinases under pathophysiological conditions, leading to tau pathology through significantly elevated phosphorylated tau.

#### Tubulin

Tubulin is an essential cytoskeleton component responsible for fundamental processes, including structural support, organelle transport, and cell division. Its assembly into microtubules is facilitated and stabilized by interactions with the neuronal tau protein. α-Syn is also known to bind tubulin, and the colocalization of these two proteins has been identified in the human and rat brains [64] (Table 3). Although a study reported the effects of α-syn on induction of tubulin polymerization [65], a contradictory result indicated that α-syn could destabilize microtubule assembly by blocking physiological interactions between tau and tubulin [66]. Residues 60–100 of α-syn were identified as the binding site for tubulin, which could contribute to inhibiting microtubule formation [67]. Even α-syn interactions with tubulin exerted different conformational effects on microtubule polymerization. Monomeric α-syn had no effect on microtubule polymerization, but tau-promoted microtubule assembly was inhibited by both protofibrils and α-syn fibrils [54]. In addition, treatment of oligomeric α-syn in dopaminergic neurons hampered tubulin polymerization and decreased mitochondrial function [68]. Taken together, interactions of α-syn with tubulin mainly affect the inhibition of microtubule assembly, and α-syn may be involved in pathogenic mechanisms rather than in normal physiological functions.

**Table 3:** Interaction between α-synuclein and tubulin

Types of ligands	Types of α-syn	Consequences
Tubulin	α-syn	α-syn aggregation↑ [64] Tubulin polymerization↑ [65] or ↓ [66, 67]
	α-syn monomer	No effect on tubulin polymerization [54]
	α-syn oligomer	Tubulin polymerization↓ [68]
	α-syn fibril	Tubulin polymerization↓ [54]

*α-syn* alpha-synuclein

The overall functions of α-syn, as a partner of Aβ, tau, and tubulin, were depicted in Fig. 3. This schematic figure focused on the AD pathogenesis induced by involvements with α-syn. On the other hand, the interactions between α-syn and these proteins could be expanded to concomitant pathology. Converse to the accelerated pathology of Aβ and tau by α-syn, synucleinopathies in patients with AD could be initiated by α-syn aggregation due to interaction with proteins. For instance, patients with DLB and PD with dementia would be involved with aberrant aggregations of Aβ and α-syn, leading to the co-existence of senile plaques and Lewy bodies in the brain [69]. Various mixed types of neurodegenerative disorders could be interpreted as causing result in the specific pathology by promoting mutual aggregations of Aβ, tau, and α-syn.

### Genetic association of α-synuclein in AD

*SNCA*, which encodes α-syn, was the first gene discovered in a patient with PD [70]. Most *SNCA* mutations are associated with PD pathology, and the involvement of AD-related genes as a genetic risk factor is limited. Interestingly, *SNCA* polymorphism of the GG frequency (rs10516846) was significantly increased in patients with AD compared to healthy control (HC) [71]. In addition, α-syn levels in CSF of patients with early-onset AD (EOAD) were higher in GG (rs10516846) carriers than in AA carriers [71], suggesting an association between *SNCA* gene polymorphisms and elevated α-syn levels in AD pathophysiology. Peripheral leukocytes in patients with AD showed elevated mRNA expression of *SNCA* with a reduced methylation rate in the intron 1 part of *SNCA*, one of the methylation regulatory regions of *SNCA* [72].

Previous studies have suggested associations between α-syn and representative genes in AD, such as amyloid precursor protein (*APP*), presenilin 1 (*PSEN1*), and apolipoprotein E (*APOE*). α-Syn and phosphorylated α-syn-positive dystrophic neurites have been observed in the brains of APP transgenic mice [73]. Additional overexpression of mutated *PSEN1* in *APP* transgenic mice accelerated Aβ-induced synucleinopathies and further promoted phosphorylation of α-syn [73]. Human studies also showed a high frequency (50–60%) of Lewy body pathology in familial AD groups, supporting an association between AD-related genes and α-syn [6, 74]. Intriguingly, α-syn pathology has been observed in the amygdala in over 90% of patients with autosomal dominant AD in *PSEN1* [75]. Direct interactions of α-syn with the presenilin 1 protein were identified in the brain tissues of cognitively normal individuals. They were significantly increased in the tissues of patients with AD and DLB with *PSEN1* mutations [76]. Increased interactions of the presenilin 1 protein with α-syn in *PSEN1*-mutated cell lines were associated with increased membrane binding and α-syn accumulation [76]. Additionally, a study revealed that cells with *PSEN1* mutations associated with AD and DLB had exacerbated phosphorylation and accumulation of α-syn [77]. In a recent CSF biomarker study, the tau/α-syn ratio was altered in patients with AD, and in particular, changes in EOAD were statistically higher than those in late-onset AD [63]. Furthermore, CSF α-syn levels were higher in symptomatic autosomal dominant AD mutation carriers than in non-mutation carriers [78]. Considering the high contribution of genetic factors in EOAD, changes in CSF α-syn levels could be influenced by AD-related genes, resulting in greater changes in autosomal dominant AD. *APOE* encodes the apolipoprotein E protein, an important lipid-binding protein for intercellular lipid redistribution in the CNS, which is a major risk factor for late-onset AD (after the age of 65 years). The possible pathophysiological roles of *APOE* and α-syn have been investigated, mainly in relation to PD. The apolipoprotein E protein level was elevated by over fourfold in transgenic mice with the α-syn A30P mutation, and *APOE* knockout in α-syn A30P transgenic mice increased the survival rate, delayed behavioral symptoms, and decreased neuronal degeneration and Aβ aggregates [79]. In particular, the APOEε4-expressing PD mouse model, but not ε2 and ε3, showed increased α-syn pathology and astrogliosis and impaired behavioral ability with worsened neuronal and synaptic loss [80]. In patients with PD, apolipoprotein E was elevated in the CSF with an abundance in dopaminergic neurons of the substantia nigra from postmortem brain tissues [81]. Although the association between the apolipoprotein E protein and α-syn has been focused on in patients with PD, it may be involved in AD pathophysiological conditions. For instance, laboratory studies have shown elevated CSF α-syn levels in APOEε4-carrier patients with AD [63, 78]. Additionally, elevated CSF α-syn levels were significantly associated with Aβ plaque burdens in APOEε4-positive individuals with autosomal dominant AD [78]. Thus, these studies described the associations of the APOEε4 risk allele with CSF α-syn levels and Aβ deposition in AD. Although the exact mechanism has not been clearly elucidated, accumulating evidence suggests that α-syn is strongly associated with AD-related genes and contributes to disease progression at the genetic level.

### CSF α-syn in AD

#### Meta-analysis of CSF α-syn in AD

**Fig. 4:**
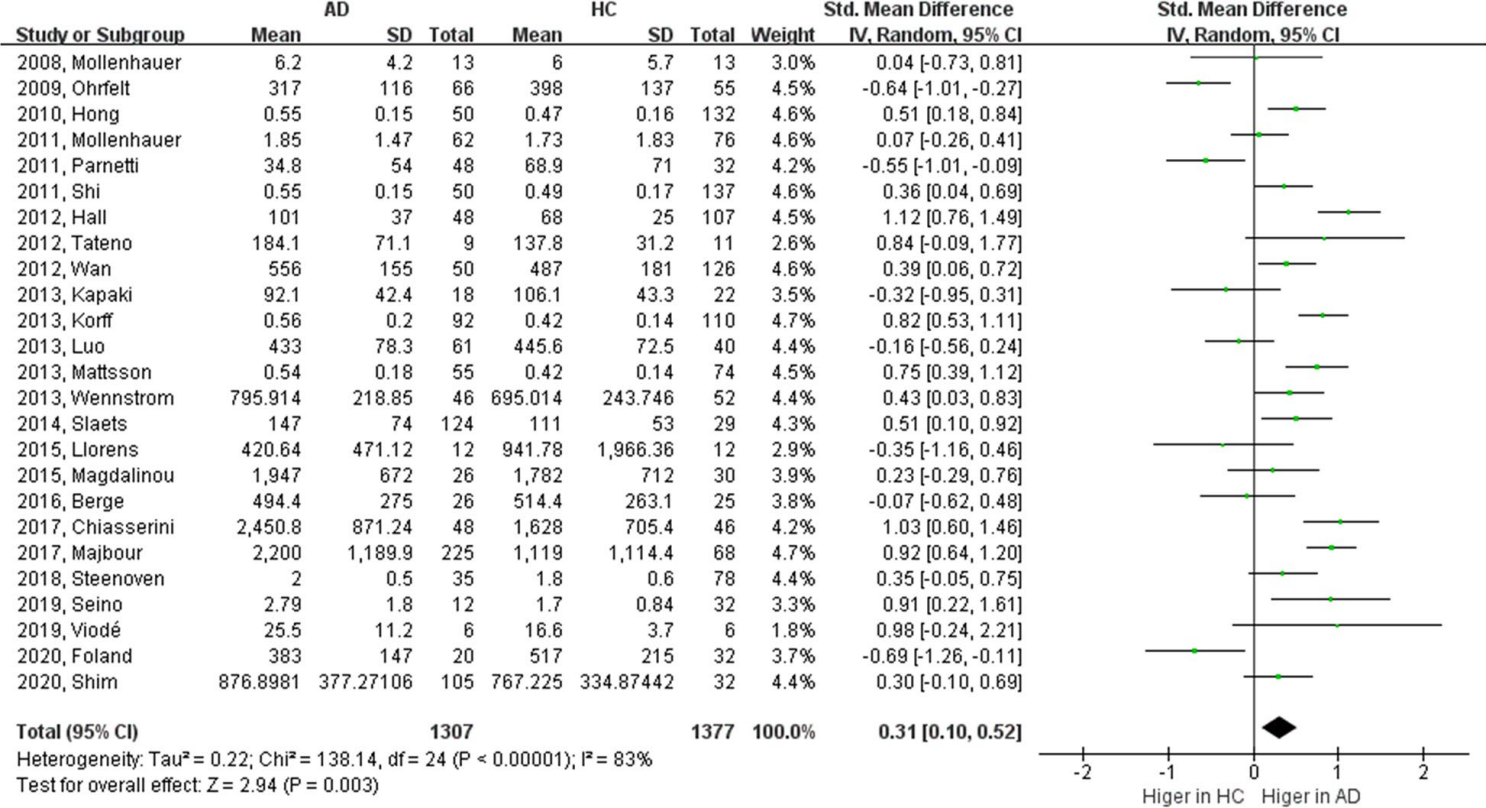
Meta-analysis of the cerebrospinal fluid α-synuclein level in Alzheimer’s disease and healthy controls CSF, cerebrospinal fluid; α-syn, alpha-synuclein; AD, Alzheimer’s disease; HC, healthy controls

α-Syn is considered to be a biomarker of synucleinopathies, including PD and DLB, as it is the main component of Lewy bodies. However, several studies have investigated the diagnostic performance of the CSF α-syn level in AD. In the meta-analysis, only the total α-syn level was considered, and the phosphorylated or oligomeric forms were excluded. We searched the PubMed and Web of Science databases to extract 38 articles related to the total α-syn level in the CSF of patients of AD. Contrary to the total α-syn level, CSF oligomeric and phosphorylated α-syn levels at serin 129 were consistently elevated in patients with PD [82, 83] but unchanged in those with AD [84]. Since two α-syn species may dominantly affect synucleinopathies rather than AD-related pathology, we speculated that total α-syn mostly of the monomeric form would be suitable for the AD diagnosis. Sixteen studies revealed statistically significant increases α-syn levels in the CSF of patients with AD [78, 84–98], whereas a few reports showed reduced levels [99–101]. In particular, α-syn levels in the CSF were significantly elevated in patients with AD with all positive CSF triple markers (Aβ_42_, total tau, and phosphorylated tau) [63, 84]. However, no difference in CSF α-syn concentrations between AD and HC were found in a few studies [63, 102–119]. Inconsistencies across studies may have resulted from misdiagnosis, co-existence of other neurodegenerative disorders, hemolysis, anticoagulant upon collection in plasma, pre-analytic sample handling, technical errors, and particularly platform differences of measurements [119]. To verify changes in CSF α-syn levels in AD, a meta-analysis was conducted with 25 studies in which a normal distribution could be obtained (Fig. 4). In addition, we excluded articles, which were sub-classified groups through additional parameters, such as CSF analysis, positron emission tomography (PET), or longitudinal observation. A total of 25 studies reported that CSF α-syn levels were statistically higher in patients with AD than in HC (*Z* = 2.94, *p* = 0.003).

Most studies revealed strong positive correlations of CSF α-syn with tau and phosphorylated tau [63, 84, 92–95, 115–117], whereas one study revealed a negative correlation [100]. Whether elevated α-syn levels in the CSF are associated with a causal or protective mechanism in AD is unknown. Considering the high correlation between tau and α-syn levels, synaptic destruction might increase the release of α-syn into the CSF, similar to tau released by neuronal death [18]. In contrast, studies showing reduced CSF α-syn levels in AD suggested lower α-syn secretion from synaptic loss [101]. The correlation between tau and α-syn levels may be associated with the causative mechanisms of AD pathology rather than the consequence of synaptic disruptions or neuronal death. NFL is a representative biomarker released into biofluids as a result of neuron destructions. It is significantly elevated in most neurodegenerative disorders, even in tauopathies and synucleinopathies [120]. In contrast, CSF α-syn levels in patients with tauopathies and synucleinopathies revealed no difference or reduced concentrations in comparison with the HC group [121–125]. A longitudinal study showed that α-syn levels in CSF were reduced in manifested and prodromal patients with PD and slightly declined after 36 months, reflecting no association of reduced CSF α-syn levels with dopaminergic neurodegeneration [126]. These studies supported that elevated α-syn levels in AD were not simply a consequence of synaptic degeneration or neuronal deaths. According to the aforementioned in vitro study, tau phosphorylation increased as the α-syn/tau ratio increased in CSF, which was proven in a previous clinical CSF study [63]. Tau and α-syn levels showed strong positive correlations in the CSF of all groups, including AD, PD, and HC. Furthermore, as the tau/α-syn ratio in the CSF had a strong correlation with the phosphorylated tau rate, tau phosphorylation was modulated according to the ratio of the two proteins. Further studies on different equilibrium states in tau and α-syn in the CSF according to the status of AD pathophysiology could provide important implications for understanding the role of α-syn in AD.

#### CSF α-syn in the early stage of AD

In previous studies, the highest α-syn levels in CSF were measured in patients with mild cognitive impairment (MCI), showing its involvement in the early stages of AD [91, 96, 117, 127]. The α-syn levels in CSF gradually increased from normal to MCI stages and then decreased in the proceeding stage in AD [78]. In addition, the α-syn levels in CSF were elevated in patients who converted from MCI to AD and with a shorter duration of AD progression [78, 95, 115]. CSF α-syn levels in patients with MCI with stable symptoms not progressing to AD were similar to those of the HC group [78]. These results suggested that α-syn perform pathological functions in AD by rapidly increasing in the period of progression from MCI to symptomatic stages. Similar to tau, CSF α-syn levels were positively associated with brain Aβ plaque deposition in the early stages of AD [78, 118]. Among individuals with subjective complaints of memory dysfunction, CSF α-syn levels in the amyloid PET-positive group tended to be higher than those in the PET-negative group [118]. In addition, the total tau/α-syn ratio in the CSF was highly concordant with CSF Aβ_42_ and amyloid PET in our study [63]. These clinical study results are consistent with the aforementioned evidence that α-syn could synergistically and directly induce Aβ aggregation in animal models. Taken together, it could assume that α-syn is associated with Aβ-related pathophysiological mechanisms in the early stages of AD.

#### Incorporation of CSF α-syn with other biomarkers

Incorporation of α-syn in CSF with other biomarkers would have better diagnostic performances. Toledo et al. proposed that patients with elevated phosphorylated tau181 and reduced α-syn levels in CSF could be classified as AD with concomitant Lewy body pathology [92]. Another study reported a differential diagnosis for patients with DLB and AD using combined α-syn and phosphorylated tau181, since the levels of both proteins were reduced in patients with DLB but elevated in patients with AD [93]. The total tau/α-syn ratio was the highest in the CSF of patients with AD [63, 100, 101] and the lowest in the CSF of patients with PD [100]. The incorporation of α-syn with triple CSF markers (Aβ, total tau, and phosphorylated tau181) revealed the best discrimination value between AD and HC and improved differential diagnosis with other neurodegenerative diseases [63, 117]. Although the utility of α-syn as a biomarker for the diagnosis of neurodegenerative diseases remains controversial, the incorporation of CSF α-syn may improve the diagnostic performance in AD and aid in the discrimination of AD from other neurodegenerative diseases. α-Syn may offer an opportunity to overcome the limitations of triple CSF biomarkers.

#### α-Syn seed amplification assay (SAA) in AD

SAA has been used to detect minimal amounts of misfolded prion in Creutzfeldt-Jakob disease. SAA has recently been expanded to the field of synucleinopathies by detecting misfolded α-syn in the CSF, olfactory mucosa, submandibular gland, skin, and brain [35, 128–134]. α-Syn SAA distinguished synucleinopathies (PD and MSA) remarkably from other pathological diseases, such as AD, amyotrophic lateral sclerosis, Pick’s disease, corticobasal degeneration, and progressive supranuclear palsy [130, 132, 135]. In particular, among patients with Lewy body-related pathology, limbic/neocortical pathology cases had high positivity in CSF and frontal cortex brain homogenate but lower positivity in AD/ALB, indicating the possibility of the discriminating ability of mixed pathologies with α-syn SAA [136]. Similar to the total α-syn level in CSF, α-syn SAA could be applied to the differential diagnosis of patients with AD with Lewy body-related pathology (particularly AD/ALB cases).

### Blood α-syn in AD

Several studies have reported attempts to diagnose AD by measuring total α-syn levels in the blood of patients with AD. Serum levels of α-syn in patients with AD showed no significant difference from those in HC [137]. In addition, there was no discernible intergroup variation in the plasma α-syn levels between AD and HC [96]. In patients with amnestic MCI, plasma α-syn levels increased throughout disease progression and had a discriminatory capacity to indicate the risk of cognitive deterioration [138]. In a recent study, AD and HC had significantly different plasma α-syn levels [139]. Elevated plasma α-syn levels in AD were positively correlated with urinary AD-associated neuronal thread protein but not with serum lipids [139]. Inconsistent results might be owing to various factors, including technical protocols, pre-analytic processes, medications, and particularly hemolysis. Since erythrocytes were the major source of α-syn, the analyzed α-syn levels could be influenced from the released of α-syn from cytosol upon hemolysis [91, 140]. Oligomer or phosphorylated α-syn levels in serum, plasma, and red blood cells were elevated and could be used to diagnose PD or MSA. However, no study has reported the two α-syn species in AD [141–144]. Although the possible changes of α-syn levels would be present in patients with AD, blood α-syn as a biomarker may not be sufficient in the current stage and should be studied further.

## Conclusions

Numerous reports have shown that α-syn is deeply involved in the pathophysiology of AD. Nevertheless, it has not been recognized in the fields of its involvement in AD mechanisms, inter-related biomarkers, or drug development. Similar to other intrinsically disordered proteins, such as Aβ, tau, and prion, α-syn can easily adapt to its diverse structures depending on the environment, wherein each conformation may influence different mechanisms. Elevated α-syn levels in AD could facilitate Aβ oligomerization, tau phosphorylation, activation of kinases, dissociations of tau and tubulin, and tau aggregation. Furthermore, the association of α-syn with genetic factors, such as *APP*, *PSEN1*, and *APOE*, could accelerate AD pathology. As a biomarker, the CSF α-syn levels were the highest in MCI, particularly in rapidly progressing patients to AD. Elevated CSF α-syn levels were also correlated with Aβ depositions in the asymptomatic stage, indicating potential applications in the early diagnosis, as a sensitive indicator of disease progression along with changes in Aβ species, including oligomeric forms. Remarkably, the incorporation of CSF α-syn with other biomarkers had strong potential for the accurate diagnosis of AD and its discrimination from other similar neurodegenerative disorders. Combined CSF α-syn with triple biomarkers could improve diagnostic and prognostic performances. Accurate early identification of AD progression using α-syn and Aβ species may help develop novel therapeutics or better treatments for patients, considering the α-syn mechanism in AD. To date, α-syn has mainly been investigated in synucleinopathies. Further studies on α-syn in AD pathophysiology would contribute to the understanding of its mechanism in AD and other neurodegenerative diseases.

## Data Availability

The datasets generated during and/or analyzed during the current study are available from the corresponding author on reasonable request.

## Abbreviations

AD: Alzheimer’s disease
Aβ: Amyloid-beta
α-Syn: α-Synuclein
PD: Parkinson’s disease
DLB: Dementia with Lewy bodies
CSF: Cerebrospinal fluid
NAC: Non-amyloid-beta component
Cryo-EM: Cryo-electron microscopy
GSK-3β: Glycogen synthase kinase-3β
HC: Healthy control
EOAD: Early-onset AD
MCI: Mild cognitive impairment
PET: Positron emission tomography
